# Polarization of ILC2s in Peripheral Blood Might Contribute to Immunosuppressive Microenvironment in Patients with Gastric Cancer

**DOI:** 10.1155/2014/923135

**Published:** 2014-03-04

**Authors:** Qingli Bie, Pan Zhang, Zhaoliang Su, Dong Zheng, Xinyu Ying, Yumin Wu, Huijian Yang, Deyu Chen, Shengjun Wang, Huaxi Xu

**Affiliations:** ^1^Department of Immunology, School of Medical Science and Laboratory Medicine, Jiangsu University, Xuefu Road 301, Zhenjiang 212013, China; ^2^Institute of Oncology, The Affiliated Hospital of Jiangsu University, Zhenjiang 212001, China

## Abstract

Newly identified nuocytes or group 2 innate lymphoid cells (ILC2s) play an important role in Th2 cell mediated immunity such as protective immune responses to helminth parasites, allergic asthma, and chronic rhinosinusitis. However, the contributions of ILC2s in the occurrence and development of cancer remain unknown. Our previous study found that there was a predominant Th2 phenotype in patients with gastric cancer. In this study, the ILC2s related genes or molecules in PBMC from patients with gastric cancer were measured, and the potential correlation between them was analyzed. The expression levels of ROR**α**, GATA3, T1/ST2, IL-17RB, CRTH2, IL-33, IL-5, and IL-4 mRNA were significantly increased in patients, but no significant changes were found in ICOS, CD45, and IL-13 expression, and there was a positive correlation between ROR**α** or IL-13 and other related factors, such as ICOS and CD45. The increased frequency of ILC2s was also found in PBMC of patients by flow cytometry. In addition, the mRNA of Arg1 and iNOS were also significantly increased in patients. These results suggested that there are polarized ILC2s in gastric cancer patients which might contribute to immunosuppressive microenvironment and closely related to the upregulation of MDSCs and M2 macrophages.

## 1. Introduction

Gastric cancer is the second most frequent cause of cancer-related death worldwide and the prognosis remains gloomy because of its high recurrence and metastasis [1]. Immunocytes have long been recognized as a factor promoting antitumor immunity, but recent study shows that it also participates in the formation of tumor immune microenvironment; the underlying immune basis remains largely mysterious [2].

Recent researches have identified several previously unrecognized distinct members of innate lymphoid cells (ILCs) family. ILCs are categorized into three groups based on the cytokines that they can produce and the transcription factors that regulate their development and function [3–5]. Group 2 ILCs are dependent on GATA-binding protein 3 (*GATA3*) [6, 7] and retinoic acid receptor-related orphan receptor-*α* (ROR**α**) for their development and function [8]. Human ILC2s are, therefore, defined by (1) various cell surface markers (i.e., IL-7R*α*, CD161, CRTH2, ICOS, and CD45); (2) the expression of receptors for the cytokines IL-33 (T1/ST2) and IL-25 (IL-17RB); or (3) their production of the ‘‘type 2” cytokines IL-13, IL-5, and IL-4 [3, 5, 9]. As we all know, the immune system plays a dual role in cancer development: it can attack tumor cells; meanwhile, it also can promote tumor growth by providing an immunosuppressive environment [10]. In our previous study, we found that there was a predominant Th2 phenotype in patients with gastric cancer [11], while several researches have demonstrated that newly identified ILC2s promote and induce the development of CD4^+^ Th2 cell-dependent immunity [12]. Thus, we proposed a hypothesis that there may be ILC2s in peripheral blood which closely related to immunosuppressive microenvironment in gastric cancer. In this study, we analyzed the frequency of ILC2s and their related factors in PBMC from patients with gastric cancer and investigated the relationship between ILC2s and the development of gastric cancer, accumulating the new information for further research on immune status in patients with gastric cancer.

## 2. Methods

### 2.1. Patients and Specimens

50 patients diagnosed newly with gastric cancer from February to May 2013 at the Affiliated People's Hospital of Jiangsu University were included in the study, 12 females and 38 males, ranging in age from 27 to 76 years (average age, 60.2 years). All the patients were untreated for their condition at the time of blood collection. The diagnosis of gastric cancer was based on commonly accepted clinical and laboratory criteria. 30 healthy volunteers were studied simultaneously as control, including 10 females and 20 males ranging in age from 30 to 70 years (average age, 51.5 years). This study was approved by the Ethical Committee of the Affiliated People's Hospital of Jiangsu University, and written informed consent was obtained from all individuals.

Peripheral blood samples were collected before surgery and plasma was frozen at −80°C immediately after centrifugation for future use. Peripheral blood mononuclear cells (PBMCs) were obtained by standard Ficoll-Hypaque density centrifugation (Tianjin Hao Yang Company, China) and then with 1ml Trizol (Invitrogen, USA) and stored at −80°C for extracting total RNA.

### 2.2. RNA Extraction and Quantitative Real-Time PCR

Total RNA was extracted by guanidinium thiocyanate phenol chloroform method and total RNA (500 ng) was reverse-transcribed using PrimeScript RT Reagent Kit Perfect Real Time (TaKaRa, Dalian, China), according to the manufacturer's instructions. On the basis of Genebank sequences, the primers used in this study were designed by Premier 5.0 software and synthesized by Shanghai Invitrogen. All sequences of primers are shown in Table 1. Quantitative real-time PCR (qRT-PCR) was conducted in a SYBR Premix Ex Taq (TaKaRa, Dalian, China) according to the manufacturer's instructions. Fold changes in the expression of each objective gene relative to *β*-actin were calculated based on the threshold cycle (Ct). All samples were performed in triplicate.

### 2.3. Flow Cytometric Quantification of ILC2s

ILC2s population was defined as Lin^−^ICOS^+^IL^−^17RB^+^. Heparinized venous blood was freshly obtained from gastric cancer patients or healthy volunteers. PBMCs were isolated by standard Ficoll-Hypaque density centrifugation (GE Healthcare) and stained with the following antibody mix: FITC-conjugated anti-human CD2, CD3, CD14, CD16, CD19, CD56, and CD235a (eBioscience, USA); Allophycocyanin- (APC-) conjugated anti-human ICOS and Peridinin-Chlorophyll Protein Complex- (PerCP-) conjugated anti-human IL-17RB (R & D, USA). The isotype control antibody was used in all cases. For FACS phenotype analysis, data were acquired on an Accuri C6 (BD company) and analyzed with FlowJo software (TreeStar, Inc.).

### 2.4. ELISA for Plasma Cytokines

Plasma levels of IL-13 (Shanghai ExCell Biology, China), IL-5 (eBioscience, USA), Arg1 (R & D, USA), IL-33, and IL-25 (Shanghai Hushang Biotechnology, Shanghai, China) were measured by ELISA kit following the manufacturer's protocols. All samples were measured in triplicate, and the mean concentration was calculated from the standard curve.

### 2.5. Statistical Analysis

All statistical analyses were performed using GraphPad Prism Version 5.0 software (San Diego, CA, USA). Data are expressed as the mean ± SD in the figures. Comparisons between groups were performed using the unpaired student's *t*-test. Pearson's correlation was used to test correlation between two continuous variables. *P* < 0.05 was considered to be statistically significant.

## 3. Results

### 3.1. Enhanced Expression Levels of *RORα* and *GATA3* in PBMC from Patients with Gastric Cancer

**Figure 1:**
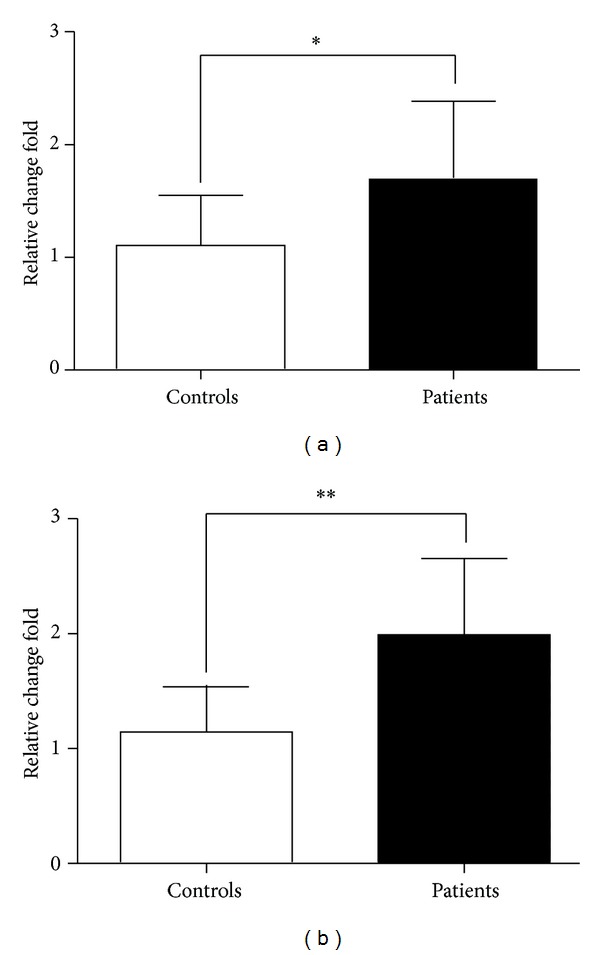
The levels of ROR**α** and GATA3 mRNA in PBMC. qRT-PCR analysis of ROR*α* (a) and GATA3 (b) mRNA levels in PBMC from gastric cancer patients and healthy controls. Data shown are represented as mean ± SD (all samples were measured in triplicate). *P < 0.05 and **P < 0.01.

The transcription factors *ROR*α** and *GATA3* are essential for the development and function of human ILC2s. To analyze the level of ILC2s in the patients with gastric cancer, the transcription factors *ROR*α** and *GATA3* mRNA in PBMC were detected. As shown in Figure 1, the mRNA expression levels of *ROR*α** and *GATA3* were significantly increased in patients with gastric cancer compared with healthy controls.

### 3.2. Increased ILC2s Relatively Specific Receptors and Surface Markers in PBMC of Patients with Gastric Cancer

**Figure 2:**
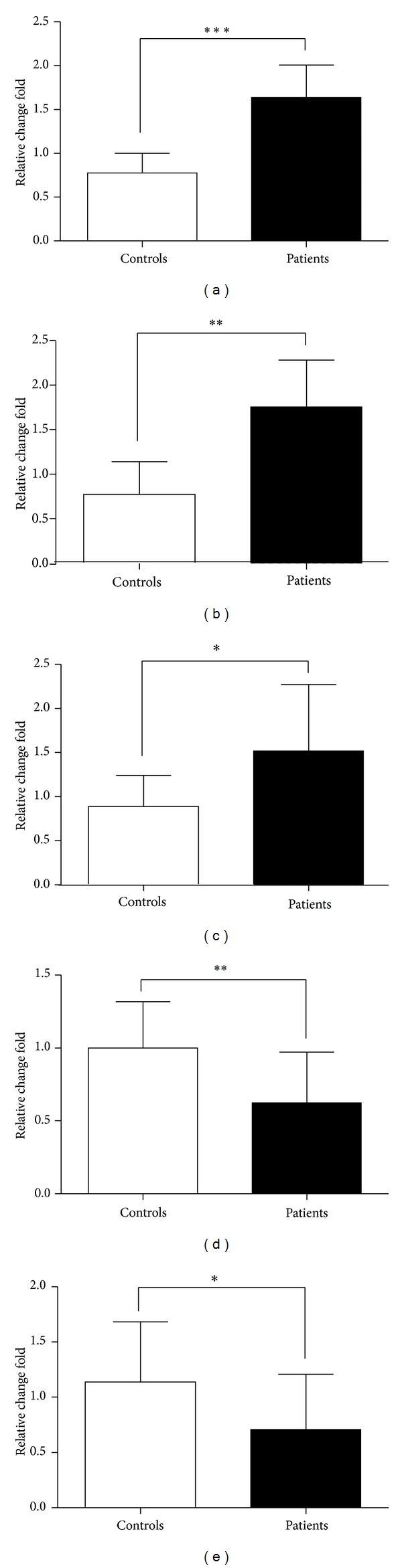
The levels of ILC2s-associated receptors and surface markers. T1/ST2, IL-17RB, CRTH2, ICOS, and CD45 mRNA in PBMC from gastric cancer patients were detected by qRT-PCR. The results of receptors analysis: T1/ST2 (a) and IL-17RB (b); the surface markers: CRTH2 (c), ICOS (d), and CD45 (e). Data shown were represented as mean ± SD (all samples were measured in triplicate). *P < 0.05, **P < 0.01, and ***P < 0.001.

ILC2s express relatively specific receptors and surface markers including T1/ST2, IL-17RB, chemoattractant receptor expressed on Th2 cells (CRTH2), inducible T cell costimulator (ICOS), and CD45. We compared the mRNA levels of the relatively specific receptors and surface markers in PBMC from patients with those from healthy controls, and the results showed that the expression of T1/ST2, IL-17RB, and CRTH2 was significantly increased in gastric cancer, while the levels of ICOS and CD45 mRNA were significantly decreased (Figure 2).

### 3.3. Different Expression Levels of Th2 Associated Cytokines in PBMC or Plasma of Patients with Gastric Cancer

**Figure 3:**
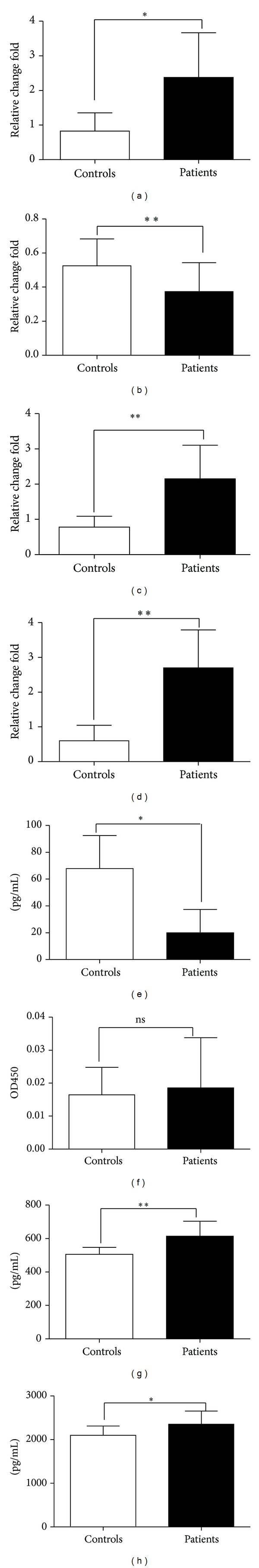
Enhanced levels of type 2 cytokines in gastric cancer. The qRT-PCR analysis of IL-33 (a), IL-13 (b), IL-5 (c), and IL-4 (d) mRNA levels in PBMC from gastric cancer patients and healthy controls; ELISA analysis of IL-13 (e), IL-5 (f), IL-33 (g), and IL-25 (h) protein levels in plasma. Data shown are represented as mean ± SD (all samples were measured in triplicate). *P < 0.05 and **P < 0.01; ns means no significance.

IL-33 or IL-25 can induce human ILC2s polarization, and ILC2s provide an innate source of T helper type 2 cytokines including IL-13, IL-5, and IL-4. In this experiment, the qRT-PCR was used to analyze the levels of IL-33, IL-13, IL-5, and IL-4 mRNA in PBMC, and ELISA was performed to evaluate the levels of these signature cytokines in plasma. Our data indicated that, both in mRNA or protein expression levels, IL-5, IL-4, IL-33, and IL-25 were significantly increased in patients, while the level of IL-13 mRNA or protein was decreased (Figure 3).

### 3.4. The Correlation between the mRNA Levels of ROR*α* and ILC2s Relatively Specific Receptors or Surface Markers and Signature Cytokines

**Figure 4:**
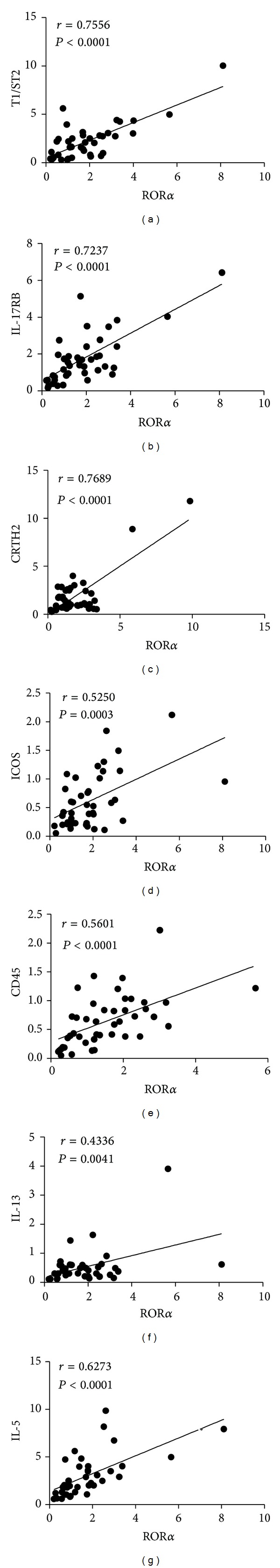
Correlation between the transcription factor ROR*α* and related genes mRNA levels. (a) The correlation of ROR*α* and T1/ST2 mRNA expression (*r* = 0.7556, *P* < 0.0001), (b) ROR*α* and IL-17RB (*r* = 0.7237, *P* < 0.0001), (c) ROR*α* and CRTH2 (*r* = 0.7689, *P* < 0.0001), (d) ROR*α* and ICOS (*r* = 0.5250, *P* = 0.0003), (e) ROR*α* and CD45 (*r* = 0.5601, *P* < 0.0001), (f) ROR*α* and IL-13 (*r* = 0.4336, *P* = 0.0041), and (g) ROR*α* and IL-5 (*r* = 0.6273, *P* < 0.0001) in gastric carcinoma patients. There were positive correlations between them.

The transcription factor *ROR*α** is essential for ILC2s development. To understand the relationship of ROR*α* and ILC2s-related markers and signature cytokines in patients with gastric cancer, we analyzed the correlation between ROR*α* and T1/ST2, IL-17RB, CRTH2, ICOS, CD45, IL-13, and IL-5 in mRNA levels. The data indicated that there was a positive correlation between ROR*α* and ILC2s-associated receptors or signature cytokines in cancer patients, respectively (Figure 4).

### 3.5. Increased Frequency of ILC2s in the PBMC of Patients with Gastric Cancer

**Figure 5:**
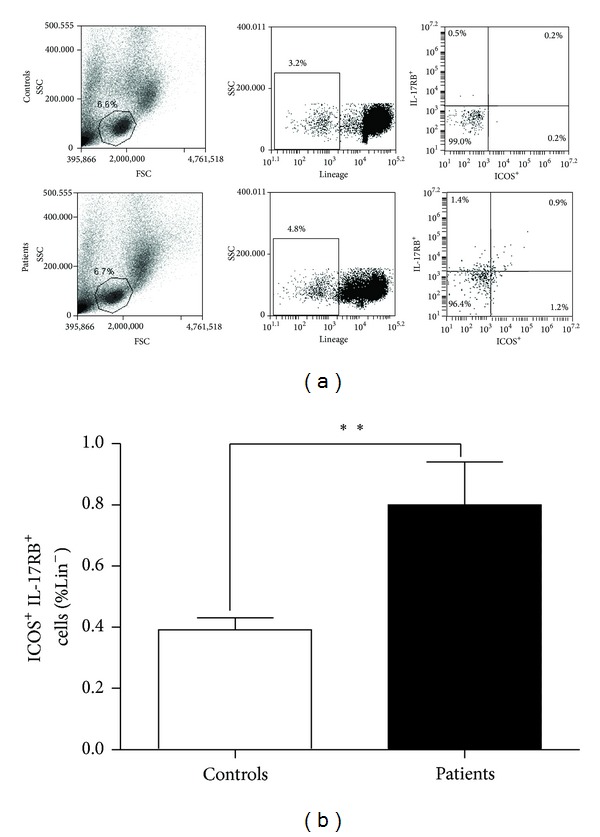
Enhanced ILC2s frequency in the PBMC from gastric cancer patients. The frequency of ILC2s in PBMC was analyzed by flow cytometry. (a) Representative diagrams of flow cytometry analysis for circulating ILC2s; (b) the frequency of ILC2s in PBMC from patients with gastric cancer was significantly increased compared with healthy controls (*n* = 30, *P* < 0.01).

The frequency of ILC2s in PBMC from gastric cancer patients or healthy volunteers was also determined by flow cytometry analysis. As shown in Figure 5, the frequency of ILC2s (Lin^−^ICOS^+^IL^−^17RB^+^) was significantly elevated in gastric cancer patients compared with healthy volunteers (*P* < 0.01).

### 3.6. Enhanced MDSCs or M2 Related Molecules in Gastric Cancer

**Figure 6:**
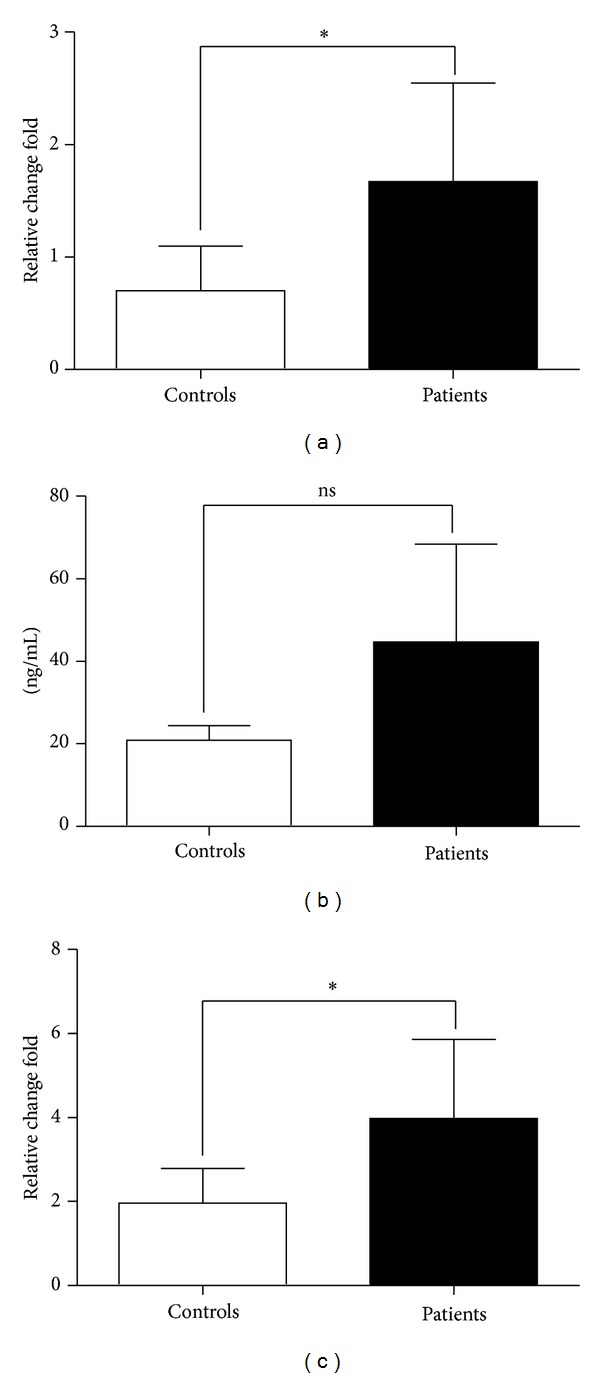
Increased expression levels of Arg1 and iNOS in gastric cancer patients. qRT-PCR analysis of Arg1 (a) and iNOS (c) mRNA levels in PBMC from gastric cancer patients and healthy controls and ELISA analysis of Arg1 protein levels (b) in plasma. Data shown were represented as mean ± SD (all samples were measured in triplicate). *P < 0.05; ns means no significance.

Arginase1 (Arg1) and inducible nitric oxide synthase (iNOS) are two different but related enzymes that are expressed highly in MDSCs [13, 14]. Furthermore, Arg1 is a well recognized marker of M2 macrophages. Several studies have certified that MDSCs and M2 macrophages are associated with immunosuppression in the tumour microenvironment [15, 16]. In this study, we examined the expression levels of Arg1 and iNOS mRNA in PBMC and Arg1 protein in plasma from patients. The data showed that the mRNA levels of Arg1 and iNOS were significantly increased and Arg1 protein was increased with no statistical significance, compared with healthy controls (Figure 6).

### 3.7. The Correlation between Activation Related Genes ICOS and CD45 and IL-13 mRNA Levels

**Figure 7:**
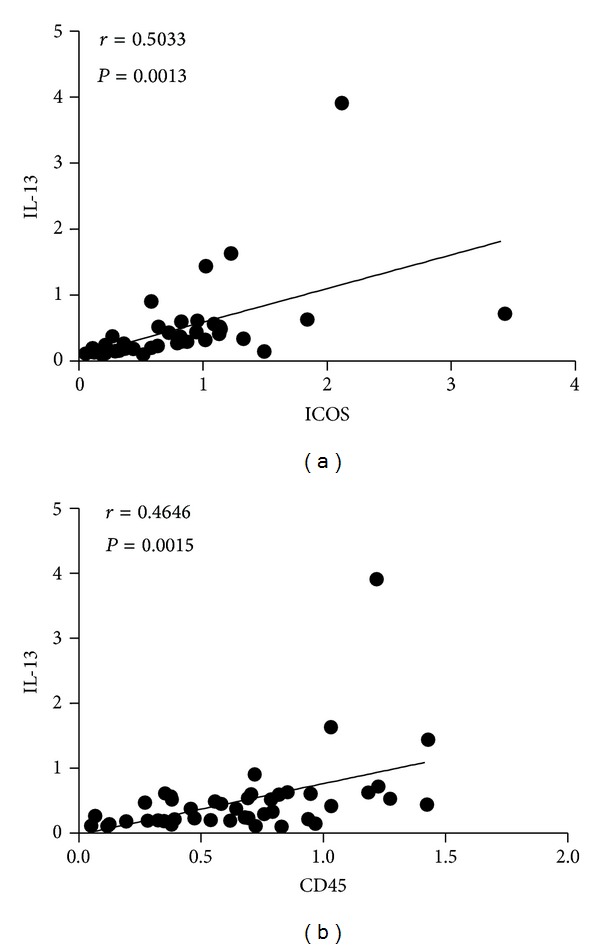
Correlation analysis between IL-13 and ICOS and CD45 mRNA levels. (a) The correlation of IL-13 and ICOS mRNA expression (*r* = 0.5033, *P* = 0.0013) and (b) IL-13 and CD45 mRNA expression (*r* = 0.4646, *P* = 0.0015) in gastric cancer patients; there was a positive correlation between them, respectively.

The correlation between the mRNA levels of IL-13 and ICOS and CD45 was analyzed, respectively, and the results demonstrated that there was a positive correlation between IL-13 and ICOS and CD45 in patients with gastric cancer (Figure 7).

## 4. Discussion

The recent identification of previously unrecognized group 2 innate lymphoid cells (ILC2s, also called nuocytes, innate helper cells, or natural helper cells) has provided new insights into our understanding of the cellular mechanisms that lead to the development of CD4^+^ Th2 cell-dependent immunity and/or inflammation at mucosal sites. This population is activated by IL-25 and/or IL-33 and expresses high levels of T1/ST2, IL-17BR, IL-7R*α*, ICOS, and CD45 [17, 18]. ILC2s represent the predominant early source of IL-13 and IL-5 to promote and induce the development of Th2-type adaptive immune response including host resistance against parasitic helminth, airway inflammation, and airways hyperreactivity in a murine model of allergic asthma and are essential in the repair of damaged respiratory tissue following acute infection with influenza virus [12, 19–24].

It has been demonstrated that CD4^+^Th1 cells and CD8^+^cytotoxic T lymphocytes (CTLs) are major mediators of antitumor immunity, while immune deviation toward Th2 will suppress Th1 development [25]. A recent study identified that ILC2s were additional cellular sources of Arg1 in the lung using an Arg1 reporter mouse and IL-33 can regulate Arg1 activity in the lung by increasing ILC2s numbers and by activating macrophages [26, 27]. In our previous study, we found that there was a predominant Th2 phenotype in patients with gastric cancer [11], and this was closely related with polarization of MDSCs and M2 macrophages [28], which indicated that immunosuppressive microenvironment was maintained by interaction between Th2 cells, MDSCs, and M2 macrophages. In view of this, we analyzed the frequency of ILC2s and the expression levels of ILC2s-related factors in PBMC of patients with gastric cancer in the present study. Our data showed that the mRNA expression levels of the transcription factors *ROR*α** and *GATA3*, the receptors T1/ST2 and IL-17RB, surface markers CRTH2, and type 2 cytokines IL-33, IL-5, and IL-4 were significantly increased in PBMCs from patients with gastric cancer compared to healthy controls. The flow cytometry analysis showed an enhanced frequency of ILC2s (Lin^−^ICOS^+^IL^−^17RB^+^) in PBMCs of cancer patients. The correlation analysis showed that there was a positive correlation between ROR*α* and T1/ST2, IL-17RB, CRTH2, ICOS, CD45, and signature cytokines IL-13 and IL-5 of ILC2s in patients. Meanwhile, the mRNA expression levels of Arg1 and iNOS which reflected the level of MDSCs or M2 macrophages were significantly increased in patients with gastric cancer. Accordingly, there may be a close relationship between MDSCs/M2 and ILC2; although IL-13 level detected in this study does not support this conclusion, it needs to be further studied.

However, the levels of ICOS or CD45 mRNA and cytokines IL-13 expression in mRNA or protein level had no significant change, even lower than the healthy control. Furthermore, there was a positive correlation between IL-13 and ICOS and CD45 in gastric cancer. ICOS was mainly expressed in activated T lymphocytes and CD45 expressed in all leukocyte; they were associated with lymphocyte activation [4]; the lower levels of these molecules might be due to some lymphocytes in a inactivated state, which involved immunosuppressive microenvironment in gastric cancer patients, and the decreased IL-13 level was thought to be responsible for the low expression of ICOS and CD45. It also indicated that the increased ILC2s in gastric cancer may be imperfect polarized ILC2s, which showed a few cytokines secreted dysfunction.

In conclusion, the enhanced ILC2s was found in gastric cancer patients through the detection of specific transcription factors, receptors, and associated cytokines and frequency of circulating ILC2s, which might participate in Th1/Th2 imbalance and related to immunosuppressive microenvironment created by MDSCs and M2 macrophages. However, the ILC2s found in the gastric cancer in this study should be partially differentiated or imperfect polarized ILC2s. It made us realize the heterogeneity in differentiation of homologous cell in different environments and thus caused the diversity of biological function. Further study will contribute to a more comprehensive understanding of ILC2s and their role in the microenvironment in gastric cancer.

## Figures and Tables

**Table 1:** The primer sequences for RT-PCR.

Gene	Sequence (5′-3′)	Accession
GATA3	Fwd: TTGTGGTGGTCTGACAGTTC	NM_002051
	Rev: AGTACAGCTCCGGACTCTTC	

ROR*α*	Fwd: CTGACGAGGACAGGAGTAGG	NM_134261
	Rev: GTGCGCAGACAGAGCTATTC	

IL-17RB	Fwd: CTTGGTGGCCTTCAACAAGC	NM_018725′
	Rev: AGAGCCGACCGTTCAATGTG	

T1/ST2	Fwd: AGATGAGTCACTGGCATACG	NM_016232
	Rev: GAGAGGCTGGCTGTTGTATT	

ICOS	Fwd: GGCATGAGAATGGTCCAAGT	NM_012092
	Rev: CATGAAGTCAGGCCTCTGGT	

CD45	Fwd: GTGAGGCGTCTGTACTGATG	NM_002838
	Rev: ACGGCTGACTTCCAGATATG	

IL-13	Fwd: GGCTGAGGTCTAAGCTAAGG′	NM_002188
	Rev: GACAGCTGGCATGTACTGTG	

IL-5	Fwd: ACTCTCCAGTGTGCCTATTC	NM_000879
	Rev: CTGCTGATAGCCAATGAGAC	

Arg1	Fwd: CAAGAAGAACGGAAGAATCAGC	NM_001244438.1
	Rev: TTGTGGTTGTCAGTGGAGTGTT	

iNOS	Fwd: CTTTCCAAGACACACTTCACCA	NM_000625.4
	Rev: TATCTCCTTTGTTACCGCTTCC	

CRTH2	Fwd: CCTCTGTGCCCAGAGCCCCACGATGTCGGC	NM_004778
	Rev: CACGGCCAAGAAGTAGGTGAAGAAG	

IL-33	Fwd: TGACGGTGTTGATGGTAAGATG	NM_033439.3
	Rev: ACAGAGTGTTCCTTGTTGTTGG	

IL-4	Fwd: GACATCTTTGCTGCCTCCA	NM_000589.3
	Rev: TACTCTGGTTGGCTTCCTTCA	

*β*-Actin	Fwd: TGGCACCCAGCACAATGAA	XM_005249820.1
	Rev: CTAAGTCATAGTCCGCCTAGAAGCA	
